# The Texas Medical News’ “Explanation,” Etc

**Published:** 1896-08

**Authors:** 


THE JULY NUMBER of the *Texas Medical Windmill* (*News*) should be called the "Daniel edition," as it expends all its wind on us, devoting five and a half pages of its editorial space to us in an endeavor to "explain" some of the inconsistencies of the editor's position and statements; how, for instance, he came to say, "without intending to create a wrong impression;" that the Association "dispensed" with Dr. Daniel's services as secretary, when he knew such was not the case; admitting, "we thought at the time Dr. Daniel was right, and we think so now." Why then try to make it appear he was wrong and had gotten "dispensed" with? The *News* man wriggles and squirms a good deal, but seems to come out at the same hole he went in. Dr. Daniel had charitably attributed the injustice evidently done him by that mis-statement of facts to pique or haste, and the doctor would have done better to let it go at that. The long "explanation" doesn't explain.

IT IS IN keeping with the *News*' unfairness to claim that Dr. Daniel had been driven from his position that a legislature can not "transfer" a responsibility and a heavy obligation to the general government (without consulting Uncle Sam), just because, finding that the *News* could not discuss the subject temperately, and without resorting to personalities and epithets, we said we would drop it. The *News* has simply been permitted to have the last say, because it is useless to continue a discussion where there is no way to decide the question. The doctor evidently thinks that his assertion, without proof, is an effective argument—conclusive. In his presentation of the matter, his assertions were never sustained by an iota of proof, while ours were sustained by facts, and the law quoted in support of our position. In his midnight resolution, "transferring" the Texas quarantine to the United States government, so often referred to, appears this statement: "the United States government is ready and willing to pay the State for the property," notwithstanding the doctor knew that Dr. Bennett had in his possession a letter from the surgeon-general of the Marine Hospital Service informing him positively and without equivocation that the United States government had *not the money* to equip or run any additional stations, and that an appropriation by congress would have to be made before such proposition could be entertained—or words to that effect, the surgeon-general being ignorant of the fact, or losing sight of it, that it was not proposed to make any "proposition;" it was not proposed to consult Uncle Sam in the matter at all; but the Texas Legislature was to be asked to "transfer" the entire business to the general government *nolens volens.*

There is little use in continuing a controversy where one's opponent has such total disregard of facts, but presents matters in whatever shape will best serve his purpose. For instance, and by way of further illustration: In quoting Dr. Davis' letter, the *News* quotes the first part only, in which Dr. Davis says (see TEXAS MEDICAL JOURNAL for July) that there is nothing in the code applicable to the question of ethics of mixed boards, for the reason, he says, probably, that the code was made before there were any boards. The *News* quotes Dr. Davis thus far, and with characteristic unfairness fails to say that Dr. Davis clearly intimates that it is an unfortunate defect, and that if the letter of the code does not forbid a physician serving on a board with an irregular, it *ought to*; and continues (and this was the gist of his letter, and sustained us in the only point we had urged, to-wit: that it was derogatory to the dignity of the profession to ask for a mixed board, inasmuch as it involves a recognition of homeopaths as a *school of medicine*, and an admission that the regular profession is a *school of medicine* also, contrary to the fundamental principles of the code): "While I thus find nothing in the existing code of ethics prohibiting a regular physician from acting as a member of a mixed board of medical examiners, I certainly do not think it wise or proper for a physician or a medical society *to ask for*, or *in any way advocate the enactment of any law* founded on the assumption that the great body of the medical profession constitute a *school of medicine*, to be disignated as *allopathy*. First, because no such "school"

exists, and never has existed, except in the fanciful mind of Hahnemann and his followers. Consequently, its use, in the enactment of laws, is a *misrepresentation of the profession* [italics ours.—Ed.], and strongly calculated to perpetuate the popular errors of past centuries."

Why did not the *News'* editor quote this? Oh no, that is not his game.

The doctor differs with the judge on a point of law. We have all heard of the backwoods preacher who, preaching from Paul's epistles, said, "and right here, brethren, I differ with brother Paul." It is worse than waste of time (and we will waste no more on the subject) to argue with one who will persistently ignore facts, however patent. In threshing over the chaff in our windy contemporary's five and a half pages of Daniel editorial, we find this (referring to "Another Clincher," *vide* "Red Back" for July, in which we exposed the utter emptiness of the pretext that it was necessary to recognize the homeopaths as a *school of medicine* "in order to get a constitutional law passed") our irascible neighbor, who can't argue without getting mad and saying spiteful things says, * * "the doctor [meaning us] errs when he implies that the constitutionality of this feature of the law has ever been passed upon by the courts of Texas." We did not err; we did not imply. We stated that the courts of Texas *had passed upon* the law (which "discrimates" against homeopaths, inasmuch as only diplomas from colleges recognized by the American Medical Association are recognized), and had pronounced it *constitutional*. We gave the case, the date, and the page of the decision, quoting the exact words. Notwithstanding, Dr. McLaughlin disputes it. It is a matter of record (but it is not the first record he has disputed). He differs with brother Paul. Judge Fly, of the Court of Civil Appeals, says (see Texas Medical Journal for July, 1896, page 39): "*There can be no question of the constitutionality of the law. * * * We see no conflict between the statute and the constitution.*" The doctor being a better judge of the law *does* see a conflict, and begs to differ with brother Fly, as he differs with brother Surgeon-General Marine Hospital Service as to whether or not there was any money on hand to purchase quarantines, although the surgeon-general of the Marine Hospital Service had, through Bennett, virtually pledged his word of honor that there was *not*. Why argue with one who willfully ignores such facts, and loses his temper and his manners because we do not think his way? Why, he would dispute a sign-board, and contradict a time-table. "You may do very well as a piece of ornamental card board, Mr. Time-table, and may do for the marines or the Salvation Army, but you 'err' when you 'imply' that the north-bound leaves at 8:30—it doesn't leave at all," is about the doctor's style of putting it.

---

BLANCHE TRAY AND SWEETHEART are all snapping at our heels. We protest it is asking too much to expect us to notice everything that growls at us. Snap away. We don't mind what is said of us, so that our friends confine themselves to a statement of *facts*. Common decency demands this, at least.

